# The Impact of Inadequate Sleep on Overtraining Syndrome in 18-22-Year-Old Male and Female College Athletes: A Literature Review

**DOI:** 10.7759/cureus.56186

**Published:** 2024-03-14

**Authors:** Hemangi Patel, Pradeep Vanguri, Divya Kumar, Dianna Levin

**Affiliations:** 1 Sports Medicine, Nova Southeastern University Dr. Kiran C. Patel College of Osteopathic Medicine, Fort Lauderdale, USA; 2 Health and Human Performance, Nova Southeastern University, Fort Lauderdale, USA; 3 Osteopathic Medicine, Nova Southeastern University, Fort Lauderdale, USA

**Keywords:** performance, recovery, collegiate athletes, overtraining syndrome, sleep

## Abstract

Both male and female athletes experience acute fatigue and decreased performance from intense training sessions and training cycles with inadequate recovery. The concept of training with insufficient recovery time is known as overtraining syndrome (OTS). Primary stressors leading to OTS include excessive training, environmental factors, and inadequate levels of sleep. Sleep is a critical component of rest, recovery, memory, and cognitive function in collegiate athletes, known as male and female athletes between 18 and 22 years old. Collegiate athletes are more prone to inadequate levels of sleep, which can lead to elevated levels of fatigue, a lack of energy, motivation, alertness, and a weakened immune system. Additionally, inadequate levels of sleep lead to decreased glycogen stores in the body, affecting the functioning of physiological pathways. The processes of removing toxins and the release of growth hormones (GHs) are also impacted. GH is secreted as the rapid eye movement (REM) phase alternates with the non-REM phase and continues to rise until it peaks in the REM sleep stage, which is important for physical recovery, memory formation, and emotional regulation. This literature review aims to summarize current research on overtraining and the physiological changes that are present in both males and females from inadequate levels of sleep, emphasizing its importance in body homeostasis.

## Introduction and background

Sleep is an essential physiologically recurring state of mind and body distinguished from wakefulness by altered consciousness, inhibited sensory activity, and a decreased ability to react to stimuli. It is regulated by circadian and homeostatic processes. Sleep is divided into two primary sleep cycles: rapid eye movement (REM) and non-REM sleep. There are numerous benefits to sleep, including memory formation, endocrine and metabolic balance, as well as immune and restorative function, all of which are essential components for both everyday individuals and elite performers like athletes. Non-REM sleep is separated into light sleep and deep, slow-wave sleep; it is known to have localized restorative effects, particularly during slow-wave sleep in the cortex [1]. REM sleep has been speculated to have a similar role in noncortical regions; it has an important role in memory consolidation and has an association with the activation of the hippocampal/limbic circuit [1]. There is a differential role of REM versus non-REM sleep in consolidating hippocampus-dependent procedural memories in relation to declarative memories as well [1]. Non-REM sleep is associated with decreased metabolic activity and unit firing, while REM sleep is an active state with increased energy expenditure and increased activity in the pons, amygdala, and most of the cortex [1].

Sleep is a critical component of rest, recovery, memory, and cognitive function in male and female collegiate athletes 18-22 years old. Sleep patterns are known to affect an athlete’s performance. When there is sleep deprivation, reaction times are slower, and there is a profound effect on mood and cognitive functions [2]. There is evidence in the literature supporting the role of sleep among both genders of college-level athletes regarding overtraining syndrome (OTS). OTS is developed when athletes engage in excessive and/or intense training without sufficient rest and recovery. OTS is linked not only to the mishandling of training intensity but also to external factors. Various biopsychosocial elements and additional factors such as underlying medical conditions, inadequate calorie intake, diminished sleep quality or quantity, and mental health issues are likely significant contributors to its onset and warrant thorough consideration [3]. Student-athletes often experience a decline in performance, acute fatigue, and mood disturbances from intense training sessions throughout the year, especially during peak performance season. When athletes fail to recover from training, they suffer muscle fatigue. These continuous cycles of training with inadequate recovery not only result in increased stress on the body but also lead to a long-term decline in performance, otherwise known as OTS [4]. Even though athletes and coaches recognize the importance of rest days, the slow onset of OTS masks the efficacy of recovery times, so the athlete is no longer able to reach their previously attainable goals [5].

OTS takes several theories into account, given the complexity and variety of symptoms that have been seen. Although none of these theories offers a clear explanation, pathophysiology must take into consideration how cytokines, the hypothalamus, glycogen, and branched-chain amino acids play a role in sleep deprivation and affect OTS [5]. Personalized care based on the unique symptomatology is essential for addressing OTS. Due to the complexity of this illness, there is no one-size-fits-all treatment; thus, the healing process for each athlete must be customized. Plans for therapy revolve around rest, either total or relative, depending on the intensity of the symptoms. While severe cases could necessitate extended rest and might not entirely resolve, mild ones might be resolved with a few weeks of exercise [5].

OTS, which involves poor sleep habits, is also often associated with frequent infections and depression, which occur following hard training and competitions [4]. In the 2023 edition of Sleep Medicine Reviews, a meta-analysis showed a substantial overlap between depression and sleep quality in adolescents, both male and female [5]. These psychological changes hinder reaction time and change emotions, leading to decreased overall performance. Elevated levels of stress and inadequate sleep can depress the immune system as well [6]. There is a profound influence of sleep on the functions of the immune system, specifically signaling molecules of pro-inflammatory cytokines such as IL-6, IL-1, and TNF-α and NF-κB, as well as concentrations in prolactin, growth hormone (GH), and cortisol [7]. Endocrine changes that occur with sleep deprivation include decreased levels of GH and prolactin, resulting in decreased growth of muscle, tissue, and bone. The process of glycogen metabolism is present during sleep, particularly in slow-wave sleep, to replenish the glycogen stores. Furthermore, decreased metabolism of glycogen due to inadequate sleep results in decreased neuronal connections and increased muscle fatigue [8]. Athletes who experience sleep deprivation have an associated risk of injury, which increases muscle tension and decreases muscle function [6]. It is widely recognized that there are multiple factors that can impact sleep and create additional challenges for females, such as the influence of their menstrual cycle and hormonal disruptions. This literature review delves into summarizing the physiological changes from inadequate levels of sleep that are present in both male and female athletes experiencing OTS.

## Review

Materials and methods

The literature review included articles detailing the discussion of overtraining, the role of sleep, and athletes with increased load and inadequate sleep. Articles were searched within the past 10 years in PubMed, Embase, Medline, and Google Scholar to have the most updated research regarding the topic reviewed. Articles finalized excluded any case reports, proposals, inaccessible articles, were not in English, were mice studies, and did not fit the study framework. The study framework included articles focusing on sleep, overtraining, and pathophysiology. Many of the articles retrieved were out of scope and focused on minute details and in-depth pathophysiology, such as cardiac biomarkers, which was not the primary focus of this review. Other excluded articles focused on the pediatric population, bone health, and specific conditions such as hypogonadism, which was not the principle of this review article. The focus of this review is to introduce the influence of sleep on athletes as a general approach, including articles relevant to both male and female genders, and discuss potential pathophysiological mechanisms present in both males and females. The keywords used to search were “sleep,” “recovery,” “overtraining,” “injury,” “males,” “females,” “pathophysiology,” and “intercollegiate athletes.” The initial search yielded 331 articles, of which 23 were selected to be included in the final analysis based on the inclusion and exclusion criteria stated above. Additionally, two articles from the 2023 edition of Sleep Medicine Reviews were included in the final analysis.

In 2024, it was brought to our attention that there was greater research in the field of sleep and athletes. A second search was completed between 2019 and 2024 to yield the most recent literature. A total of 324 articles were found relating to sleep and athletes. However, once the criteria were specified to exclude any case reports, proposals, inaccessible articles, articles that were not in English, mice studies, and only included articles relating to sleep, overtrained athletes, aged 18-22, males and females, recovery, increased load, fatigue, and pathophysiology, eight articles were retrieved. The literature review paper focuses on introducing the impact of sleep on overtrained athletes without delving into great specifics, as many articles did.

Review

OTS

Competitive athletes are constantly pushed to their limits in terms of physical activity and training to increase their performance. When paired with adequate recovery time, such training can prove to be beneficial for an athlete’s performance. However, when athletes engage in excessive and/or intense training without sufficient rest and recovery, they can develop OTS [4]. OTS results in fatigue and overall prolonged underperformance, which can lead to career-ending injuries. OTS can also lead to a range of negative consequences, such as decreased performance, increased risk of injury, and changes in mood and behavior [9]. Many psychological and neuroendocrine factors play a role in the development of OTS.

OTS can be difficult to diagnose and present with various symptoms such as fatigue, decreased performance, altered mood, and immune system dysfunction. It is vital for athletes and coaches to be aware of the signs of OTS and to take appropriate steps to prevent it, such as properly scheduling training and rest periods, monitoring for signs of overtraining, and allowing for adequate recovery time. Treatment of OTS typically involves reducing the volume and intensity of training and addressing any nutritional or psychological issues contributing to the condition.

Diagnosis

OTS is physiologically characterized as a “sport-specific” decrease in performance together with disturbances in mood state in an individual. This underperformance persists despite a period of recovery lasting several weeks or months [9]. Currently, there is no specific diagnostic tool to identify if an athlete is experiencing OTS, so clinical evaluations and diagnosis involve the exclusion of other causes of fatigue (Table 1). A conclusive diagnosis of OTS may require hematological screenings and more extensive investigations to rule out conditions like viral myocarditis and arrhythmia [9]. In addition, the exclusion of organic diseases, e.g., iron deficiency anemia, endocrine diseases, infectious diseases, and feeding disorders (anorexia and bulimia), leads to the diagnosis of OTS [9]. Muscle-derived markers, specifically creatine kinase (CK), are common markers to evaluate the level of exercise stress and recovery present [10]. However, because CK levels are high in athletes and vary based on the type of exercise and intensity, they cannot be used as an indicator of inadequate recovery from training [9]. In addition, dysregulation of the hypothalamus and its axes, specifically the hypothalamic-pituitary-adrenal (HPA) and the hypothalamic-pituitary-gonadal axes, can account for early increases in pre- and postexercise catecholamine levels. These athletes can have drastic changes primarily in cortisol, adrenocorticotropic hormone, testosterone, and estrogen, which can increase susceptibility to upper respiratory tract infection [4]. This immunosuppression can be indicated by increased cortisol levels and low glutamine levels, which may be used as diagnostic tools [7]. In recent years, inflammatory markers, specifically CRP and IL-6, have been used to measure exercise strain and whether there is adequate recovery. When there is a return to the baseline of the inflammatory markers, it can be interpreted as a restoration of homeostasis, suggesting full recovery [10]. The combination of CRP, IL-6, and CK levels in the evaluation of an athlete can provide a better evaluation of the level of damage present from overtraining. It is important to consider that the immune system is complex, and intense training sensations and elevated levels of cortisol influence immune markers in diverse ways. Therefore, the variations can alter in everyone, and thus it is important to consider multiple diagnostic markers [11].

**Table 1:** OTS synopsis. The main factors in the diagnosis, pathophysiology, and management of OTS. OTS, overtraining syndrome

Factors	Description
Diagnosis [9]	Diagnosis of exclusion of causes of fatigue and hematological screening
Pathophysiological [13]	Increased iron levels induce oxidative stress – an increase in protein carbonyl, nitrotyrosine, and malondialdehyde levels leading to muscle damage
Management [15]	Measuring progress via monitoring tools, rest, aerobic exercise, relaxation, and psychotherapy or counseling

Pathophysiology

Studies have indicated that overtraining has been correlated with increased reactive oxidative stress, leading to impaired antioxidant defense. The first initial connection between muscle function and cellular oxidoreductive (redox) balance was established when researchers observed elevated free radicals in frog limb muscles stimulated with tetanic contractions [12]. The basic premise of redox signaling as we know it now is that reactive oxygen species (ROS) regulate reversible posttranslational modifications, including sulfenic acid, sulfoxide, and disulfide formations, S-nitrosylation, and S-glutathionylation to thiol moieties on target proteins implicated in cell signaling [12]. Reactive oxidative stress occurs when there are elevated levels of ROS, which are toxic by-products of metabolism [13]. When there are more ROS than antioxidants in the body, reactive oxidative stress occurs.

Markers of protein oxidative damage were quantified by increased concentrations of plasma protein carbonyls, nitrotyrosine, and malondialdehyde (Table 1). The study done by Tanskanen et al. indicated that an increase in plasma protein carbonyl concentrations may be linked to phagocytic cell invasion of damaged muscles, which then leads to increased oxidative species [13]. However, levels of nitrotyrosine and malondialdehyde, a lipid peroxidation indicator, did not change between groups. Although oxidative stress can be used as an accurate measure of diagnosis, other factors need to be accounted for, such as menstrual cycles in female athletes [13]. Evidence shows that estrogen can have antioxidant properties, so gender needs to be accounted for when evaluating a patient for OTS [13]. Overall, protein carbonyls are shown to be a promising indicator of increased oxidative stress in OTS, as they help determine the presence of damaged muscle [13].

Within a cell, iron is stored by ferritin to protect against free radical reactions that lead to inflammatory processes. During stressful conditions, as seen in excessive exercise, the ferritin is broken down by lysosomes and proteasomes, therefore releasing iron and promoting inflammatory feedback [14]. The data in Żurek et al.’s study regarding tennis players who participated in training camps showed that individuals had a drop in serum iron levels, resulting in reduced inflammation evident through the decline of tumor necrosis factor alpha (TNFα), a pro-inflammatory cytokine [14]. In the study, it was noted that prior to the athletes arriving at the training camps, their TNF levels were increased, suggesting a lack of balance between exercise and recovery already present. The training program provided a balance between exercise and rest, which ended up decreasing levels of TNFα, leading to overall decreased inflammation [14]. The detection limit for TNFα was 0.038 pg⋅mL^-1^ in this study.

Management

The recovery assessment involves monitoring the autonomic nervous system through heart rate variability (HRV), which is the measure of variability in heartbeats. HRV was measured at rest and after exercise while interpreting the training phase and/or load phase [15]. This method of looking at HRV has become increasingly popular due to its noninvasive, time-efficient, and inexpensive applicability to many athletes. Another variable to assess recovery is blood lactate level and markers that focus on inflammatory and stress responses in the body, such as CK, urea nitrogen, cortisol, and free testosterone [15]. The management of OTS is subjective; athletes use the rate of perceived exertion, which is how hard an individual perceives their body is working during exercise, to measure intensity and load, which coaches and trainers can use to modify the recovery process for athletes. There are many monitoring tools that can be used in conjunction with each other to determine optimal recovery for athletes, such as power output measuring devices, time-motion analysis, internal load measurements via oxygen uptake and heart rate, as well as sleep quality and quantity [15].

A holistic approach to the management of OTS consists of rest and regeneration strategies (Table 1), such as exercising aerobically for a few minutes each day and slowly building up over time, as well as cross-training to avoid increasing exercise intensity too quickly [15]. The recovery strategy differs for everyone, as there are no set guidelines. In addition to physical strategies, it may be helpful to incorporate relaxation, counseling, and psychotherapy, as well as addressing any outside stressors and any concurrent illnesses such as depression (Table 1). It is important to slowly increase the intensity of training after a period of rest and to allow for full recovery.

The Role of Sleep in Modulating Body Processes

Sleep has many influences on the body, such as recovery at the cellular level, endocrine system homeostasis and circadian regulation, energy conservation, and changes in the synaptic plasticity of the brain leading to cognitive influences [1]. Endocrine homeostasis involves various hormones that play a role in the growth of muscle, tissue, and bone [1]. In Mignot’s article, he discusses whether homeostasis and circadian regulation are linked. He hypothesizes that sleep deprivation can impair the expression of circadian genes and modulate electrical activity in the suprachiasmatic nucleus, which is a known regulator of circadian rhythms [1]. Therefore, he suggests that a division between endocrine homeostasis and circadian rhythms may not be valid and believes a link is present between endocrine system homeostasis and circadian regulation. Further, it is vital for athletes to have enough REM and non-REM sleep to consolidate memory for optimal performance in their sport. A large amount of gene expression in the brain is restored during sleep, such as through protein synthesis and replenishment of neurotransmitters, which is vital for recovery and performance in athletes [1]. When chronic sleep deprivation is present, there are alterations to cognition, memory, emotions, and the neuroendocrine pathway. It is evident that sleep is shown to have a vital influence on growth and adaptation within the body and is an important consideration for athletes to have the ability to perform at their optimal level.

Impact of Sleep on Emotions and Psychological Functions

Athletes experience unfavorable training, competition, travel, and practice times that contribute to reductions in sleep quantity and quality [16]. Athletes tend to have strict practice schedules that cause them to fall into a continuous pattern of fatigue and chronic sleep deprivation. Individuals with less than six hours of sleep showed a greater decline in alertness, cognitive accuracy, and memory than individuals who had eight or more hours of sleep a night [16]. Additionally, chronic sleep deprivation has adverse effects on endocrine and cardiovascular physiology [16]. For proper athletic performance, individuals are recommended to obtain eight or more hours of sleep. Inadequate levels of sleep have a profound impact on alertness, learning, memory, impulsivity, and executive functions [17]. Sleep-deprived individuals are shown to have an impairment in adapting to abrupt changes in reaction time during performance, which affects cognition and the feedback of information. In addition, in an experiment using a study design to investigate the effect of acute sleep loss on instrumental learning, the results indicate that sleep-deprived individuals (staying awake the entire night) have a slower reaction time and greater decline in many cognitive functions compared to sleep-control individuals (sleeping at least six and a half hours) [16]. In one experiment using a psychomotor vigilance task that assesses an individual’s sustained attention and reaction time, athletes experiencing less than eight hours of sleep had a slower reaction time [16]. The baseline reaction time level was found to be 500 ms, and athletes experiencing less than eight hours of sleep exceeded 500 ms. Athletes who slept greater or equal to eight hours had a reaction time of less than 500 ms [16]. This has a direct impact on performance due to their inability to function appropriately on the field. According to Symons et al., endurance athletes competing in triathlons, running, cycling, and swimming who experienced an increased training load had an associated decline in cognitive function [18]. Psychomotor speed decreased, suggesting impaired performance, whereas reaction time rose in both the Stroop color-word test and the reaction time test [18]. Also, impulsivity was higher in behavioral tasks, according to the studies evaluated by Symons, with overloaded athletes making a higher percentage of impulsive decisions [18].

Another important consideration in an athlete’s preparation and recovery is the influence of emotions. Athletes experience elevated levels of stress, and how they react to these levels of stress can impact their mood and sleep activity. Prior to competitions, athletes tend to have poor sleep due to high stress, which leads to mood changes and a decline in performance [8]. There are many factors that contribute to elevated levels of stress, such as performance anxiety, fear of injury, playing a new position, and the pressure of winning [19]. The negative impact of inadequate sleep on mood can affect decision-making due to the presence of heightened emotions [8]. There is an increased level of depression, stress, anxiety, frustration, anger, and lower confidence in sleep-deprived individuals, which can directly impact performance (Table 2) [8]. The presence of increased psychological stress increases the release of inflammatory mediators such as IL-6, causing a decline in the immune system [20] as well. The excessive expression of IL-6 can lead to continuously elevated levels of IL-6 in skeletal muscle, leading to reduced respiratory capacity [21]. Additionally, there is a correlation between inadequate sleep and increased fatigue, which is an important determinant of OTS that will be further discussed.

**Table 2:** Impact of inadequate sleep. Major physiological changes were found in relation to a lack of adequate sleep. GH, growth hormone

Physiological change	Cause	Effect
Immune system decline [7]	Pro-inflammatory cytokines released	Increased presence of illnesses and upper respiratory tract infections
Changes in endocrine function [25]	Decreased levels of GH and prolactin	Decreased growth of muscle, tissue, and bone
Increased risk of injury [6]	Accumulation of injuries	Increased muscle tension and decreased muscle function
Decreased restorative sleep [26]	Inability to remove toxins and modulate recovery	Neurocognitive function decline
Decreased glycogen metabolism [8]	Lack of glycogen	Decreased neuronal connection and increased muscle fatigue
Changes in psychological function [28]	Hindered reaction time and changes in emotions	Decreased overall performance

Immune System and GH Release

The imbalance between training load and recovery can play a potential role in the presence of illnesses in athletes. Excessive intensity of exercise provokes an acute inflammatory response in the body, triggering a cellular response from the immune system to remove the inflammatory triggers and the damaged tissue [10]. The goal of the inflammatory response is to restore the tissue integrity that has been compromised. When athletes experience moderate levels of stress in their training, there is an increased prevalence of injury [6]. The increased levels of stress lead to an underactive immune system [6]. A common method to improve the increased state of fatigue and enhance recovery is to get adequate levels of sleep [22]. Sleep allows for recovery in cognitive processes and metabolic functions. There is a profound influence of sleep on the functions of the immune system, specifically signaling molecules of pro-inflammatory cytokines such as IL-6, IL-1, and TNF-α and NF-κB, as well as concentrations in prolactin, GH, and cortisol [7]. Smith’s cytokine hypothesis theory describes how an imbalance between excessive exercise and inadequate recovery time leads to muscle trauma and further increases pro-inflammatory cytokine production, mainly IL-6, TNFα, and IL-1 beta [23]. This implies that frequent, intense physical activity can create a persistent intramuscular cytokine profile, like chronic inflammatory diseases such as rheumatoid arthritis, which is frequently associated with muscle weakness [21]. It is important to note that the HPA axis regulates the antigen-presenting cells, known as dendritic cells (DCs) [2]. Cortisol decreases the activation of DC, which influences the levels of IL-6, IL-12, and TNF-α [2]. During parts of REM sleep, there was a positive correlation between time spent in REM and IL-6 release, which is a crucial factor in proper immune system function [7]. In sleep-deprived individuals, the presence of illness, specifically upper respiratory infections, is increased due to the decrease in function of the immune system, such as levels of IL-6, IL-12, and TNF-α (Table 2). When there are alterations to the immune system, specifically inflammatory pathways, there is concern for worsening health in the future, such as cancer and cardiovascular diseases [20]. Thus, it is important to focus on the impact of inadequate sleep on athletes to prevent long-term complications.

OTS can reduce appetite, leading to consistent caloric deficits during training, thereby increasing stress hormone levels and cytokine production, which hinders performance. In addition, insufficient hydration results in fatigue, injury, and immunosuppression and affects cardiovascular responses. Adequate fluid consumption during exercise is needed to match the sweat loss because it is critical for sustaining physiological functions. Carbohydrate consumption during exercise not only boosts performance but also plays a distinct role in preventing dehydration. Maintaining an adequate daily caloric intake positively influences muscle recovery and hormonal balance, leading to enhanced sports performance. Optimizing nutrition, hydration, and caloric intake is pivotal for preventing OTS, facilitating muscle recovery, and maximizing athletic performance [23,24].

**Figure 1:**
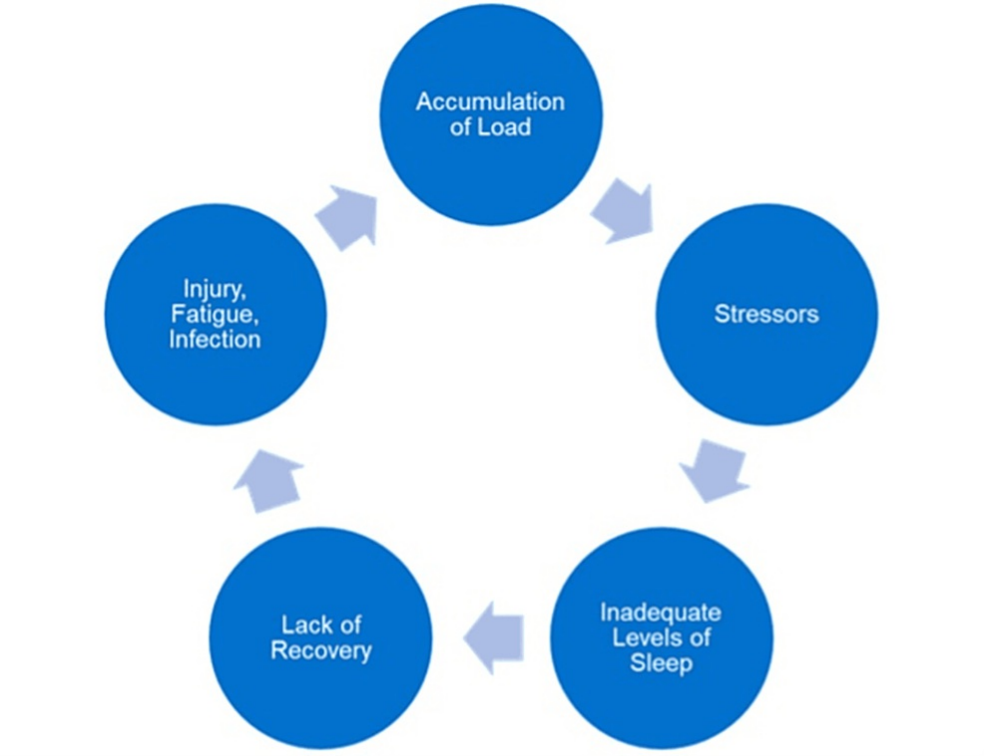
OTS cycle. A visualization of how the accumulation of load, stressors, inadequate sleep, and inadequate recovery can lead to athletes experiencing recurrent infections, fatigue, and injuries secondary to OTS. OTS, overtraining syndrome Figure created by the authors.

In relation to OTS, a state of fatigue depresses the immune system, impacting the performance of an athlete (Figure 1). Periods of high-volume training leading to fatigue and immune system decline are correlated to low levels of sleep, which further confirms the importance of sleep in the recovery processes of athletes experiencing OTS [22]. A future implication would be to evaluate the effect of napping on fatigue in athletes experiencing OTS.

Additionally, sleep causes endocrine changes such as decreasing concentrations of GH and prolactin release (Table 2). GH secretion occurs just after sleep onset and continues to rise, released during REM sleep [25]. There is also evidence of exercise inducing the release of GH, especially in high-intensity training such as sprinting, which can be augmented in athletes experiencing a lack of GH release during REM sleep due to sleep deprivation [25]. Since athletes are continuously changing their travel, competition, and practice schedules, this leads to sleep deprivation. Sleep deprivation can change the timing of when GH is released, even causing an absent release [25]. Thus, coaches may consider implementing daily high-intensity training to increase the release of GH that is deprived during inadequate sleep cycles.

Injury Prevention

It is evident that both males and females are impacted by inadequate levels of sleep, with females having more influential factors. However, both male and female athletes who experience sleep deprivation have an associated increased risk of injury. It is shown in collegiate basketball players that increasing sleep to 10 hours allowed players to sprint faster, have more accuracy in their shots, and improve their overall well-being [2]. In relation to OTS, as athletes age and experience sleep deprivation, there is potential for overuse of muscles, leading to the accumulation of injuries [2]. Athletes experiencing OTS experience high levels of stress, which can result in higher risks of injury as stress increases muscle tension in the body, causes a reduction in motor and flexibility, and increases the presence of fatigue (Table 2) [6].

To maintain a proper balance and avoid OTS, there must be a balance between stress and recovery [6]. An important consideration for athletes is reducing unwanted stress in the preseason period to reduce the incidence of injury due to overtraining during the season [6]. The preseason period is a critical period for athletes, as the study by Hamlin et al. found an increased incidence of injury and illness with a high workload during the preseason within the first seven weeks of training. After this period, even with the increased workload, there was no higher incidence of injury, exemplifying the critical role preseason plays for athletes [6].

Restorative Functions of Sleep

The brain is unique as it does not have a conventional lymphatic system [26]. Instead, the interstitial space surrounding the brain consists of β-amyloid (Aβ), α-synuclein, and tau proteins, with CSF removing proteins and toxins [26]. During the awake state, there is less space in the interstitial fluid, which has the potential to accumulate waste [26]. In the sleep state, the space is twice the size, allowing for more effective clearance of toxins such as Aβ and other degradation products that can be harmful [26]. There is a restoration function of sleep in relation to injuries, cognition, biological processes, glycogen metabolism, and the removal of toxins to allow the body to optimally function (Table 2). Furthermore, the presence of increased toxins due to inadequate sleep can play a potential role in the future development of neurodegenerative diseases [26]. The process of glycogen metabolism is present during sleep, particularly in slow-wave sleep, to replenish the glycogen stores that have been depleted during wake periods [27].

When there is sleep deprivation, there is diminished glycogen metabolism, which affects the astrocytes of the brain [27]. The process of glycogen metabolism results in lactate release that the neurons use as a source of energy to regulate the sleep-wake process [27]. With a lack of glycogen from sleep deprivation, the brain cannot properly function. Additionally, glycogen is the energy source for muscles to function properly. When there are decreased glycogen stores, muscles are not able to function properly, leading to increased fatigue, soreness, and a decline in performance.

The Impact of Sleep on Athlete Performance

As the training load increases, the amount of sleep an athlete has at night declines, leading to greater levels of fatigue and decreased performance [28]. Sleep has a key role in many components, such as learning, memory, cell growth and repair, glycogen metabolism, immune system functions, fatigue levels, and recovery [29]. There is current evidence showing athletes experiencing overtraining have less sleep quantity and quality [28]. Since fatigue levels have a direct correlation with overtraining and sleep modulates fatigue levels, it is important for athletes to have adequate sleep. There are many factors that may contribute to inadequate sleep among overtrained athletes, such as training load and environmental factors such as school stress, competition times, and training times [28]. It is prevalent that increases in training loads in an athlete cause a decline in sleep efficiency and an increased presence of fatigue [28]. With inadequate sleep levels, there is an increased risk of injury, poor performance, loss of motivation, and decreased recovery [29]. The increased training load suppresses many functions in the body, such as the immune system, causing heavy legs during sleep and soreness or pain from muscle damage due to a lack of recovery from inadequate sleep [28]. The presence of fatigue causes a lack of energy, muscle pain, decreased motivation and alertness, and changes in mood from the decreased dopamine [28]. Athletes experiencing adequate sleep release higher levels of dopamine, which decreases fatigue and the incidence of overtraining. The findings of this study suggest sleep should be a great focus during high training loads, as there is a relationship between lack of sleep efficiency and overtraining.

It is common for athletes experiencing overtraining to have increased levels of fatigue. In the study by Mah et al., athletes with 10 hours of sleep had lower fatigue scores [2]. In the study by Campbell et al., when athletes extended their sleep time by two hours for five to seven weeks, there was a significant improvement in performance and fatigue, confirming the importance of adequate sleep with training [28]. These findings support the idea that increased levels of sleep can directly decrease the prevalence of OTS in athletes. Additionally, reaction time is a vital component of successful performance. It is evident that extending sleep to 10 hours a night resulted in a faster reaction time, which led to an improvement in performance [2,30]. Another crucial factor in performance is the psychological state, particularly the mood of the athlete. Athletes experiencing a sleep extension of 10 hours a night had an increase in mood and self-perception, which correlates with the enhancement of performance [2,30].

Further, an important consideration in coaching is to use a tapering strategy before a competition, which is explained as a load decrease in training sessions before a competition to allow proper recovery time [31]. The tapering strategy resulted in athletes experiencing less fatigue and increased sleep quality and performance [31]. The tapering strategy should be investigated further as it plays a role in improving athletes’ levels of fatigue to prevent overtraining. There is also an important consideration of the number of competition days per week. The study presented by Clemente et al. found that having two basketball matches a week in comparison to one resulted in lower sleep quality and greater levels of fatigue despite lower training loads [31]. This finding suggests an accumulation effect of high training loads in the body, resulting in greater fatigue and muscle soreness [31]. A future implication of this study would be to focus on one match versus two matches per week and the effect on perceived fatigue and overtraining.

It is a common assumption that eight hours of sleep is considered adequate for healthy brain and body function. In the study of Belenky et al., the minimum amount of sleep to satisfy brain function, performance, and recovery was found to be four and a half hours a night, per Horne’s hypothesis. However, recovery continues to increase as the hours of sleep increase past four and a half hours. If sleep is lower than four and a half hours a night, there is a continual degradation in performance [32]. If individuals experience chronic sleep restriction of less than seven hours consistently, the brain undergoes changes to sustain performance and prevent injury while causing persistent changes in neurotransmitter modulation and gene expression that can cause potential detrimental effects in the future [32]. Even though the brain can functionally adapt to lower levels of sleep, as presented by Horne’s hypothesis, it is evident that athletes require eight or more hours of sleep to retain cognitive and physiological functions and perform at their optimal level while simultaneously reducing the chances of developing OTS.

Discussion

OTS is a condition of fatigue and underperformance that can lead to a decline in physiological and emotional function. Fatigue is most commonly a result of a lack of sleep due to an athlete’s intense practice schedule. Sleep deprivation for an athlete does not allow the athlete to adequately recover, which can further lead to cognitive dysfunction, changes in mood, and increased daytime sleepiness. Athletes can also experience slow reaction times, alter psychomotor abilities, and decrease physical performance during their careers.

Individuals who experience sleep deprivation are shown to have a decline in their immune function, cognitive abilities, ability to metabolize toxins, and modulation of endocrine functions such as cortisol and GH release. Athletes have a continuous cycle of training with minimal time to sleep due to balancing their sport with education and social factors. Inadequate sleep habits in athletes can cause the decline of many body functions in relation to neuroendocrine and psychological functions and increase fatigue levels. The state of increased load with decreased recovery time is defined as OTS.

As stated by Irwin et al., when athletes experience a lack of sleep, they have an altered immune system that can lead to chronic conditions such as cancer and cardiovascular disease, as well as less severe situations such as upper respiratory tract infections [20]. The decline in immune function leads to a chronic state of fatigue, which can then contribute to OTS. The presence of inadequate sleep further declines the immune system and can cause further physical complications in athletes, such as tender muscles, frequent episodes of underperformance, and injuries present in OTS.

When athletes suffer from a lack of sleep, they experience decreased glycogen metabolism and impaired brain function. This influences an athlete’s efficiency by decreasing speed, agility, and reaction time. This can then lead to a wide range of psychological complications due to decreased levels of dopamine, such as a player’s frustration with their own performance, decreased alertness, and a decline in their mood.

The presence of continuous sleep deprivation is common among athletes due to rigorous training schedules. Inadequate sleep levels can lead to declines in alertness, cognitive accuracy, and memory, as well as adverse effects on neuroendocrine functions such as cardiovascular physiology, glycogen metabolism, and cortisol production.

There are a few solutions to decrease the chance of developing OTS. Athletes’ schedules should allot appropriate time for sleep. This includes maintaining sleep hygiene, such as turning off screens early, avoiding alcohol and heavy meals before sleep, and taking hot showers before sleeping. Secondly, athletes should have rest days where they are not engaging in high-intensity exercises but can participate in light activities, such as walking and jogging. The rest day allows the body to repair and recover from prior high-intensity days, avoiding the development of OTS. Lastly, athletes should have mandatory check-ins with a wellness coach or psychiatrist to assess their well-being. Overall, athletes should be monitored physically, mentally, and nutritionally to address concerns about OTS.

## Conclusions

Sleep deprivation contributes to fatigue and underperformance, which define OTS. Athletes who undergo rigorous training without proper periods of rest can experience changes to their bodies ranging from altered moods to physical deterioration. Scientifically, a lack of sleep can diminish glycogen stores in an individual, which is necessary for proper brain function, cause a decline in the immune system, and cause psychological disturbances. Furthermore, the impact of sleep deprivation can lead to decreased agility, speed, and reaction time, increasing injuries. Although it is known that there are additional factors that influence females, such as the menstrual cycle and hormonal changes, this literature review aims to focus on the factors influencing both males and females. Further research should focus on developing diagnostic tools and markers for OTS, which can further assist physicians in making an accurate diagnosis. In addition, having coaches identify early signs of OTS by increasing awareness may help athletes in the longevity of their careers. If future studies can identify definitive markers in OTS, athletes may be able to undergo consistent testing to diagnose and treat OTS prior to career-ending damages.
